# *α*4*β*7 Integrin Antagonist Vedolizumab for the Treatment of Refractory Ileitis

**DOI:** 10.1155/2019/2091089

**Published:** 2019-04-22

**Authors:** Rafał Filip, Błażej Goliat, Małgorzata Dziechciaż, Piotr Dąbrowski, Michał Osuchowski, Piotr Paluszkiewicz

**Affiliations:** ^1^Department of Gastroenterology with IBD Unit of Clinical Hospital 2, University of Rzeszow, Lwowska 60, 35-301 Rzeszow, Poland; ^2^Department of Health Sciences, Health Care Institute of PWSTE, Czarneckiego 16, 37-500 Jaroslaw, Poland; ^3^Department of Rheumatology of Clinical Hospital 2, University of Rzeszow, Lwowska 60, 35-301 Rzeszow, Poland; ^4^Department of Pathology of Clinical Hospital 2, University of Rzeszow, Lwowska 60, 35-301 Rzeszow, Poland; ^5^Department of Gastrointestinal Surgery, Institute of Haematology and Transfusion Medicine in Warsaw, Indiry Ghandi 14, 02-776 Warsaw, Poland

## Abstract

**Background and Aims:**

Ulcerative colitis (UC) is a superficial diffuse inflammation restricted to the colon and rectum. Inflammation within the small bowel may occur several years after a pancolectomy. The pathogenesis is unknown and seems to be different from Crohn's disease or other causes of diseases of the small intestine, but an association with colectomy due to UC is very likely.

**Methods and Results:**

We describe the case of a patient with a probable diagnosis of pan-UC accompanied by Sjögren's syndrome and partial IgA deficiency, who developed enteritis after a restorative pancolectomy. For induction and maintenance of remission, the patient was successfully treated with mycofenolate mofetil (MMF) and vedolizumab (VDZ).

**Conclusions:**

We suggest that a previously refractory to standard therapy UC-related enteritis can be treated with combination MMF and VDZ.

## 1. Introduction

Proctocolectomy is the method of choice in refractory ulcerative colitis (UC) [1]. Occasionally, the previously unaffected small bowel can be affected following a colectomy for UC. The so-called ‘UC-related pan-enteritis', characterised by diffuse inflammatory changes within the small bowel, was documented in several case reports [2]. Systemic corticotherapy is the method of choice for induction of remission, but different options for maintenance therapy, including mesalamine, cyclosporine, azathioprine, and biologics, have also been described up to now [2]. However, due to the limited amount of data, standardised treatment strategies for UC-related enteritis have not been established yet. In this report, we describe the case of a patient with previous diagnosis of refractory steroid-dependent pancolitis (pan-UC) accompanied by Sjögren's syndrome and a partial IgA deficiency, who developed enteritis after a restorative pancolectomy. We also describe the results of a novel treatment approach with the use of combined mycofenolate mofetil (MMF) and vedolizumab (VDZ).

## 2. Case Report

### 2.1. A History of IBD and Partial IgA Deficiency

A 20-year-old female was initially diagnosed at the age of 13 (2010) with pan-UC. In the following years, she was treated with mesalamine, corticosteroids, cyclosporine, and azathioprine; however, no satisfactory clinical or endoscopical response was observed. Corticosteroid-dependent disease despite immunosuppressive therapy led to the initiation of infliximab in 2011. The response was positive, but the treatment was terminated after the 3^rd^ dose due to anaphylaxis. Thereafter adalimumab was introduced; however, the patient did not respond to the therapy. From the beginning (2010), extraintestinal manifestations of IBD, especially from the joints and skin, occurred; therefore, the patient was also under rheumatologist supervision with the diagnosis of reactive arthritis and leukocytoclastic vasculitis. In the same time period, due to persistent isolated (although detectable) IgA deficiency, partial IgA deficiency was also diagnosed.

### 2.2. Surgery due to Pan-UC

In 2012, the patient was referred to a surgery unit with the intention of proctocolectomy with ileal pouch-anal anastomosis (IPAA). However, due to uncertain nature of the disease, a total abdominal colectomy (TAC) and ileal-rectal anastomosis (IRA) were performed. 

The postcolectomy histopathology revealed fulminant active chronic inflammatory bowel disease, fully in keeping with active chronic ulcerative colitis involving the whole colon; spontaneously visible deep ulcerations might eventually suggest indeterminate colitis or CD. Although the terminal ileum appeared normal, the j-pouch was not formed. Repeated serology was again negative for both perinuclear antineutrophil cytoplasmic (p-ANCA) and anti-*Saccharomyces cerevisiae* antibodies (ASCA).

### 2.3. History of Sjögren's Syndrome

In 2013, the patient was hospitalised in the Rheumatology Department where Sjögren's syndrome was recognised with a typical clinical (xerostomia, xerophthalmia, lymphadenopathia, and inflammation of the parotid glands), serological (specific antinuclear antibodies), and histological (labial glands biopsy) features. Initially, due to severe joint complaints, methotrexate was introduced to the treatment. Because of abdominal side effects, methotrexate was withdrawn and chloroquine added.

### 2.4. Postcolectomy Ileitis

In February 2017, the patient was admitted to our department due to severe anemia, increased fatigue, and anxiety. She also complained of abdominal and anal pain; her bowel frequency was 20 times during the day and 5-6 times at night, passing urgent loose stools. An endoscopy showed diffuse edematous inflamed mucosa within the ileum, ileorectal anastomosis, and bleeding inflammatory changes in the rectal stump.

Pathology of the ileal biopsy revealed mixed, acute, and chronic inflammatory infiltration of moderate grade. Her symptoms poorly responded to an empirical course of ciprofloxacin with metronidazole and topical hydrocortisone.

**Figure 1:**
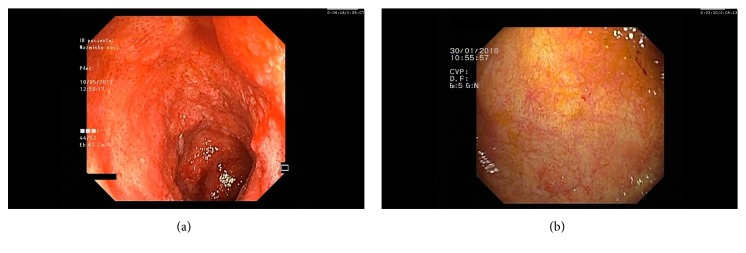
Representative endoscopy images: (a). Initial ileoscopy showing erythematous, edematous friable mucosa with the presence of spontaneous bleeding. (b). Follow-up ileoscopy demonstrating normal looking mucosa after 5 months of vedolizumab therapy.

**Figure 2:**
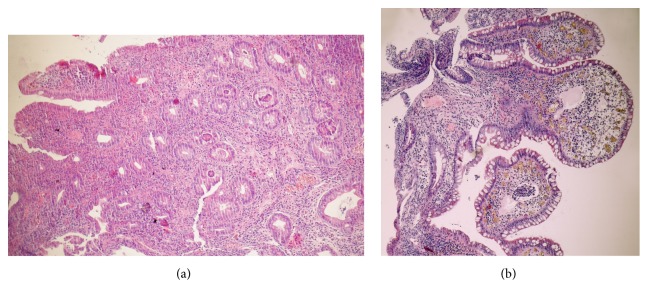
Representative histology sections of the ileum. The biopsy specimen before the treatment (a) reveals villous architectural distortion and a combination of acute and chronic abundant inflammation of the lamina propria. The inflammation consists of neutrophils, plasma cells, and lymphocytes. After 5 months of vedolizumab therapy (b), stromal oedema, and hyperaemia only a few lymphocytes and plasma cells in the lamina propria are visible.

Two months later a follow-up endoscopy showed progression of inflammatory changes—diffuse edematous inflamed mucosa with small superficial aphthous-like lesions and superficial bleeding visible within the whole examined ileum [Figure 1(a)]. The pathology specimen showed typical features of UC [Figure 2(a)]. In MR enterography no CD-like or other lesions were found. In the meantime, again negative results of viral pathogens, celiac serology, and antibodies to enterocytes and stool cultures were obtained. UC-related pan-enteritis was suspected and the decision of systemic steroid dose escalation up to 40 mg of methylprednisolone iv was made. However, no satisfactory symptom resolution was achieved; therefore, GI cut-off followed by parenteral nutrition (TPN) was initiated and she was discharged with an oral methylprednisolone taper and mesalamine.

**Figure 3:**
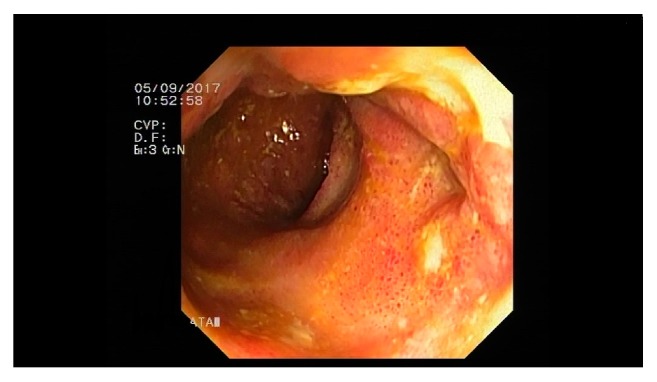
Representative endoscopy image of rectum and ileorectal anastomosis with swelling changes, congestion, and numerous aft-like loss of mucous membrane.

After three months of home TPN, the patient was admitted to hospital due to abdominal aggravation of symptoms followed by more than 20 bloody stools per day. Surprisingly, in the pelvic MR imaging, the presence of rectovaginal fistula was visualised. An endoscopy revealed active mucosal inflammation within the ileum, ileorectal anastomosis, and also rectal stump [Figure 3]. In addition, the patient had symptoms of active arthritis. Based on the history of previous azathioprine, cyclosporine, methotrexate, and infliximab intolerance, MMF in a daily dose of 1500mg was initiated. A follow-up visit revealed no clinical nor endoscopic improvement. What is of importance here, apart from MMF, was that the patient was still treated with low doses of oral steroids, mesalamine, and chloroquine. In the meantime, the patient was also advised to undergo surgical treatment including end-ileostomy, but due to consent refusal, the surgery was not performed. Therefore, VDZ in a standard dose of 300mg intravenously was introduced to the therapy. After the 3^rd^ dose of induction treatment, a significant reduction in the number of stools (<10 per day) and less rectal bleeding was observed, but at this time no endoscopic improvement was noticeable. However, the next endoscopy scheduled ten weeks later revealed significant endoscopic improvement [Figures 1(b) and 2(b)]. Moreover, the patient reported significant fistula improvement. Currently, the patient is in steroid-free remission of enteritis and arthritis on MMF and continuing maintenance therapy with VDZ.

## 3. Discussion

After restorative colectomy, inflammatory changes within the small intestine usually indicate Crohn's disease (CD); however, the ileum can also be affected by other entities like prepouch ileitis, a continuous inflammation of the retained ileum [3]. Although it is histologically different from CD, it is often mistaken as CD on an endoscopy [4, 5]. Data are also available on rare cases of extensive enteritis in patients who passed a proctocolectomy without j-pouch formation. In these particular cases, both the endoscopy and the histology reveal a diffuse mucosal inflammatory process, which is typical of UC involving the small bowel [6, 7]. In our patient, the disease in the small bowel was mimicking UC and did not appear to respond to increased doses of corticosteroids nor to three-month GI cut-off followed by TPN. But what is most interesting here is that this is also a case of rare poly-autoimmunity. Since Sjögren's syndrome and ulcerative colitis are both relatively common conditions, their coexistence with IgA deficiency was exclusively reported in 1996 in only one patient [8]. There is some evidence suggesting that the occurrence of poly-autoimmunity may affect autoimmune disease severity [9]. One could speculate that reduced mucosal defense in IgA deficiency could be a predisposing factor to chronic submucosal inflammation in the ileum and, due to immunostimulation, might also add to the lack of satisfactory response to applied therapies, including corticosteroids and immunosuppression. Other diagnostic considerations normally include an infectious, ischemic, toxic, or immunological cause, but all these were excluded.

Anyway, during the whole observation period, CD was mostly considered in this setting, even if the endoscopic and histopathological changes were typical of ulcerative colitis, and the pathological changes in biopsies showed no granulomas. Diagnosis of CD was justifiable since it is known that only 10–12% of patients with known* de novo* CD had granulomas on mucosal biopsy in nonpouch patients [10] and* vice versa*; up to 20% of patients who passed the IPAA can also develop a CD-like phenotype of the pouch, characterised by inflammation in the afferent limb [11]. Moreover, late onset of rectovaginal fistula also indicates the presence of CD. On the other hand, however, one could also speculate that this fistula might also be the result of prolonged inflammation of ileoanal anastomosis, similar to pouch fistulas. Nevertheless, based on the consolidated results of the radiography, endoscopy, pathology, and the clinical manifestations, we primarily took into account the diagnosis of the postcolectomy ileitis.

The pathogenesis of UC-related pan-enteritis is unknown. Given the low incidence, only case reports have been published [2]. Data suggesting which therapy should be administered in these patients when antibiotic, corticosteroid, or immunomodulators therapy fails are also limited. After approval of VDZ in 2015, a few patients with post-IPAA pouchitis were switched to anti-integrin therapy alone or in combination with other drugs and showed a sustained clinical and endoscopic response [12–15]. In our patient, various combinations of antibiotics, steroids, mesalazine, and probiotics were introduced to treatment with no or only transient response. Therefore, we decided to include VDZ 300 mg intravenously in a standard dosing regimen. After four doses, the ileal inflammation almost completely cleared, and this was also accompanied by significant reduction of abdominal symptoms.

Conclusions are that UC-related enteritis is a rare condition with distinct endoscopic and histological inflammation that resembles the inflammation in the colon in patients with UC. Novelties of our case include its successful combined therapy with MMF and VDZ. Although no side effects were observed, more data regarding the safety profile are needed. Furthermore, future research is required for better understanding of the pathogenesis of UC-related enteritis as well as the role of overlapping IgA deficiency.

## References

[1] Mukewar S., Wu X., Lopez R., Shen B. (2014). Comparison of long-term outcomes of S and J pouches and continent ileostomies in ulcerative colitis patients with restorative proctocolectomy-experience in subspecialty pouch center. *Journal of Crohn's and Colitis*.

[2] Hoentjen F., Hanauer S. B., Hart J., Rubin D. T. (2013). Long-term treatment of patients with a history of ulcerative colitis who develop gastritis and pan-enteritis after colectomy. *Journal of Clinical Gastroenterology*.

[3] Rottoli M., Vallicelli C., Bigonzi E. (2018). Prepouch ileitis after ileal pouch-anal anastomosis: patterns of presentation and risk factors for failure of treatment. *Journal of Crohn's and Colitis*.

[4] Bell A. J., Price A. B., Forbes A., Ciclitira P. J., Groves C., Nicholls R. J. (2006). Pre-pouch ileitis: A disease of the ileum in ulcerative colitis after restorative proctocolectomy. *Colorectal Disease*.

[5] Shen B., Fazio V. W., Remzi F. H., Lashner B. A. (2005). Clinical approach to diseases of ileal pouch-anal anastomosis. *American Journal of Gastroenterology*.

[6] Corporaal S., Karrenbeld A., Van Linde K. D., Voskuil J. H., Kleibeuker J. H., Dijkstra G. (2009). Diffuse enteritis after colectomy for ulcerative colitis: Two case reports and review of the literature. *European Journal of Gastroenterology & Hepatology*.

[7] Gooding I. R., Springall R., Talbot I. C., Silk D. B. A. (2008). Idiopathic small-intestinal inflammation after colectomy for ulcerative colitis. *Clinical Gastroenterology and Hepatology*.

[8] Steuer A., McCrea D. J., Colaco C. B. (1996). Primary Sjogren's syndrome, ulcerative colitis and selective IgA deficiency.. *Postgraduate Medical Journal*.

[9] Rojas-Villarraga A., Amaya-Amaya J., Rodriguez-Rodriguez A., Mantilla R. D., Anaya J. (2012). Introducing polyautoimmunity: secondary autoimmune diseases no longer exist. *Autoimmune Diseases*.

[10] Shen B., Fazio V. W., Remzi F. H. (2007). Clinical features and quality of life in patients with different phenotypes of crohn*ʼ*s disease of the ileal pouch. *Diseases of the Colon & Rectum*.

[11] Shen B., Remzi F. H., Brzezinski A. (2008). Risk factors for pouch failure in patients with different phenotypes of Crohn’s disease of the pouch. *Inflammatory Bowel Diseases*.

[12] Schmid M., Frick J.-S., Malek N., Goetz M. (2017). Successful treatment of pouchitis with Vedolizumab, but not fecal microbiota transfer (FMT), after proctocolectomy in ulcerative colitis. *International Journal of Colorectal Disease*.

[13] Bethge J., Meffert S., Ellrichmann M., Conrad C., Nikolaus S., Schreiber S. (2017). Combination therapy with vedolizumab and etanercept in a patient with pouchitis and spondylarthritis. *BMJ Open Gastroenterology*.

[14] Coletta M., Paroni M., Caprioli F. (2017). Successful treatment with vedolizumab in a patient with chronic refractory pouchitis and primary sclerosing cholangitis. *Journal of Crohn's and Colitis*.

[15] Segal J. P., Rottoli M., Felwick R. K. (2018). Biological therapy for the treatment of prepouch ileitis: a retrospective observational study from three centers. *Clinical and Experimental Gastroenterology*.

